# Factors associated with home delivery preference among pregnant women in Ethiopia: a cross-sectional study

**DOI:** 10.1080/16549716.2022.2080934

**Published:** 2022-07-22

**Authors:** Henok Mulatu Teferi, Miguel San Sebastian, Mazen Baroudi

**Affiliations:** Department of Epidemiology and Global Health, Umeå University, Umeå, Sweden

**Keywords:** Home delivery, preference, community, maternal health, Ethiopia

## Abstract

**Background:**

Home delivery is associated with a high risk of maternal and neonatal mortality. The prevalence and factors associated with home delivery have been studied retrospectively among women in Ethiopia. However, no national studies have assessed pregnant women’s preferences for home delivery.

**Objective:**

To assess factors associated with preferences for home delivery among pregnant women in Ethiopia.

**Methods:**

We analysed a sample of 678 pregnant women derived from the 2019 performance monitoring for action cross-sectional survey. The association between pregnant women’s preferences for home delivery and several individual, household, healthcare, and community factors were explored through log-Poisson regression with robust variance.

**Results:**

The weighted prevalence of pregnant women’s preferences for home delivery in Ethiopia was 33%. Pregnant women between the ages of 15–19 years (PR = 2.3; 95% CI: 1.43–4.00) had a higher preference for home delivery compared to those above 34 years. Those who had no Antenatal care (ANC) visit in the current pregnancy (PR = 1.5; 95% CI: 1.11–2.11), multipara women (PR = 1.8; 95% CI: 1.19–2.92) those who did not discuss place of delivery with their partners (PR = 1.5; 95% CI: 1.18–2.10), did not participate in a community-based program called ‘1 to 5’ network meetings (PR = 4.5; 95% CI: 1.09–18.95), and those who perceived low community support for facility delivery (PR = 2.2; 95% CI: 1.53–3.20) had a higher prevalence of home delivery preference compared to their references.

**Conclusions:**

A significant proportion of pregnant women preferred home deliveries in Ethiopia. Household and community supporting factors such as not discussing place of delivery with a partner, not participating in women developmental army meetings, and perceived low community support were associated with preference for home delivery. Interventions should address these factors to increase facility deliveries in Ethiopia.

## Background

Home delivery is a global public health concern, mainly in low- and middle-income countries (LMIC). A recent study showed that 3 out of 10 women in the LMICs gave birth at home [1]. The prevalence of home delivery in sub-Saharan African (SSA) countries is 34% [2], with Ethiopia as one of the countries with the highest rate. According to the latest Ethiopian demographic and health survey (EDHS) report in 2019, 51% of women delivered at home [3].

While most of the home deliveries in high-income countries are attended by a skilled health professional [4], in low-income countries such as Ethiopia, this is rarely done, which increases the risk for delivery-related complications [5]. A meta-analysis conducted in the SSA countries showed a 21% higher risk of perinatal mortality during home deliveries compared to deliveries at a health facility [6]. Moreover, a study conducted in Ethiopia showed 2.6 times higher odds of maternal mortality among women who delivered at home compared to those who delivered at a health facility [7]. A similar finding was reported by another study conducted in Cameroon [8].

Studies conducted in SSA, including Ethiopia, have explored the factors associated with home delivery. These factors can be broadly grouped into a sociodemographic, household, healthcare, and community factors. Sociodemographic factors associated with home delivery included multiparity, women’s poor socioeconomic status, place of residence, low maternal and husband education, older maternal age, lack of media exposure, and living in rural areas [9–15]. Likewise, factors associated with home deliveries included household factors, such as living in male-headed households and husband preference for home delivery, as well as healthcare factors, such as poor attitude of nurses, low antenatal care (ANC) visits, and low-quality healthcare services [10,12]. Community factors, such as belonging to pastoralist communities and communities with positive perceptions for traditional birth attendants (TBA), were also associated with home deliveries [11,13].

Although prior studies conducted in Ethiopia have retrospectively explored factors associated with home delivery in postnatal women, to the best of our knowledge, no study at the national level has focused on the determinants of preference for home delivery. Understanding the place of delivery preference is important because it might determine the likely setting of the actual birth, i.e. women who prefer to give birth at home will most likely deliver at home [16]. In addition, studying pregnant women’s preferences for place of delivery can help to measure the perception of healthcare needs during delivery, which is the first step in seeking healthcare [17].

This study aimed to assess the individual, household-supporting, healthcare, and community-supporting factors associated with pregnant women’s preferences for home delivery in Ethiopia.

## Methods

### Study setting

Ethiopia is a land-locked country located in the horn of Africa with an estimated population of 112 million [18]. Based on their characteristics such as high poverty, difficult weather conditions, and poor infrastructure, four of the nine regions (Afar, Benishangul-Gumuz, Gambela, and Somali) are regarded as emerging and they comprise 9.6% of the country’s population [19]. On the other hand, Amhara, Oromia, Tigray, South Nations Nationalities and Peoples region (SNNPs), and Harari together comprise 86.4% of the total population, and compared to the emerging regions, they have relatively developed infrastructures. Addis Ababa and Dire Dawa are the two city administrations in the country.

Since 2011, Ethiopia has implemented a Women Developmental Army (WDA) programme, which is a community-based intervention [20]. The WDA leaders are unpaid volunteers that are supported and supervised by the health extension workers (HEW) (trained government-employed community health workers which mainly provide health promotion and preventive services to the community) [20]. WDA includes structural arrangements that involve ‘1 to 5’ and ‘1 to 30’ networks [20]. A ‘1 to 5’ network involves women from six households in the same neighborhood and is led by a ‘model woman’ (a woman who practiced a lifestyle deemed healthy and development-minded by the government) selected from these households [20,21]. Five ‘1 to 5’ networks form ‘1 to 30’ networks [20,21]. The members of these networks regularly meet to discuss different health-related issues including maternal and child health and have a significant role in promoting healthy behaviours and healthcare utilization in the community [20,21].

### Data source and study design

The 2019 Ethiopia Performance Monitoring for Action (PMA) cross-sectional survey data was used for this study. PMA Ethiopia is a national representative survey conducted in collaboration with Addis Ababa University, the Ethiopian Federal Ministry of Health, and the Johns Hopkins Bloomberg School of Public Health [22]. The survey was collected by trained resident female enumerators between September–December 2019 [22]. The data includes several reproductive, maternal and newborn health indicators and are publicly available on the PMA website for research purposes.

### Sampling procedure and study population

**Figure 1.**
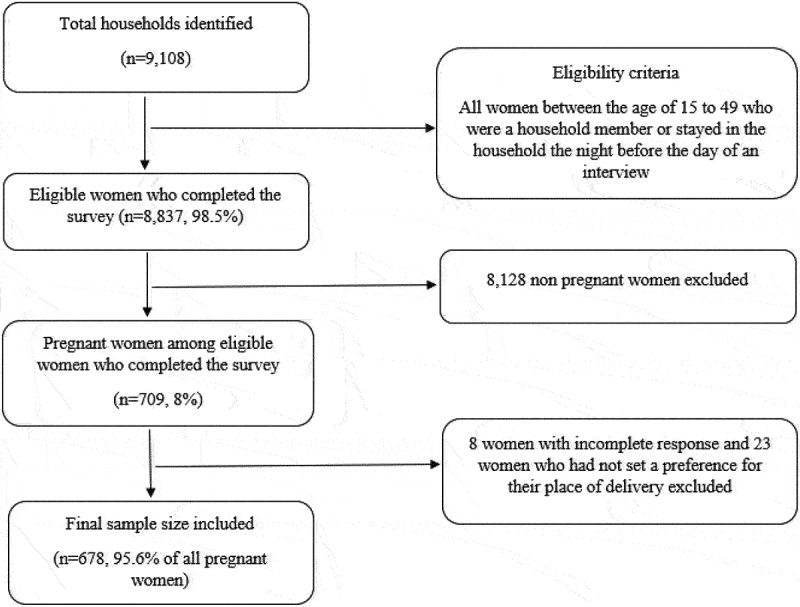
Schematic presentation of sampling procedure.

PMA Ethiopia used a two-stage clustered sampling, using the place of residence and major regions as strata. A total of 265 enumeration areas (EA) were identified and a random selection was used to select 35 households from within each EA [22]. A total of 9,108 households were identified, of which all women between the ages of 15–49 who stayed in the household the night before the day of the interview or who were members of the households were eligible [22]. A total of 8,837 women completed the ‘Female Questionnaire’ with a total response rate of 98.5% [22]. Among them, 709 (8%) were pregnant at the time of the survey and were the target population of this study. We excluded eight women who had incomplete responses, and 23 women who had not set a preference for their places of delivery. Finally, a total of 678 pregnant women were included in the analysis, accounting for 95.6% of the women who were currently pregnant in the survey sample (Figure 1).

### Measurements

#### Dependent variable

The dependent variable in this study was the desired place of delivery. Pregnant women who wished to deliver at public or private health facilities were coded as 0 (preference for facility delivery), while those who wished to give birth at their own homes or other homes were coded as 1 (preference for home delivery).

#### Independent variables

The socio-ecological model for health [23] provided the conceptual framework for this study. This model supports a holistic multilevel perspective for health and health-related behaviours and attitudes, whereby health and health behaviours occur as a result of interrelated interactions between the individual, interpersonal, social, organisational, and community factors [23]. Using the model as a guide for organising the data, factors associated with pregnant women’s preferences for home delivery were categorised into individual, household-supporting, healthcare, and community-supporting factors.

**Individual factors**: the variables included under this category were *age* (15–19, 20–34, > 34 years), *educational status* (no formal education, primary education, secondary education or higher), *place of residence* (urban, rural), *region* (emerging, developed, city administrations), *wealth index* (lowest, lower, middle, higher, highest quantile), and *parity* (nullipara: women who have never given birth, primipara: women who have given birth only once, multipara: women who have given birth twice or more). Finally, *media exposure* was assessed indirectly by whether a pregnant woman accessed information regarding family planning through radio, TV, newspaper, social media, or text messages on her mobile device. Based on this, those who accessed the information by at least one of the media outlets were categorised as ‘media exposed’ and those who did not as ‘did not have media exposure’.

**Household-supporting factors**: to capture *partner support for ANC* visits, the pregnant women were asked as ‘has your partner encouraged you to go to the clinic for ANC?’ Pregnant women who responded ‘yes, encouraged’ were categorised as having partner support. While those who responded ‘no, did not encourage’, ‘no, actively discourage’, ‘no partner’, or **‘**do not know’ were categorised as no partner support. *Discussion with a partner about the place of delivery* for the current pregnancy was dichotomised into ‘yes’ and ‘no’.

**Healthcare factor**: included one variable, *ANC visit in the current pregnancy*, which was categorsied as ‘ANC visit by skilled health professionals’ (those who had ANC visit by skilled health professionals such as medical doctors, midwives, and nurses), ‘ANC visit by HEWs’ (women who had ANC visit only by the HEWs) and ‘no ANC visit’ (women who had no ANC visit).

**Community-supporting factors**: Two variables were included in this group. The first, *participating in ‘1 to 5 meetings*’, was coded as ‘yes’ or ‘no’. To address *community support*, the women were asked ‘do most, some, few, or no people in your community encourage women to deliver at a facility?’ Those who responded: ‘most people’ were categorised as high support, while those who responded ‘some’, ‘few’, or ‘no people’ were categorised as having low support. Those who responded ‘don’t know’ were also grouped under this category.

### Statistical analysis

The individual, household-supporting, healthcare, and community-supporting factors of the participants were described using frequencies and percentages and cross-tabulated with delivery place preference. To estimate crude and adjusted prevalence ratios (PR) at 95% confidence interval (CI), we first tried log-binomial regression, but the model failed to converge while conducting the multiple regression. Therefore, based on the recommendation from Janani et. al, we decided to use log-Poisson regression with robust variance to estimate the PR [24]. The process followed a four-stage regression modeling approach: in model 1, bivariate analysis of each independent variable with the outcome variable was performed and the statistically significant variables at 5% level of significance were included in the subsequent models; in model 2, regression was performed adjusting for individual and household-supporting factors; in model 3, healthcare factors were added; and finally, in model 4, the community-supporting factors were included. Due to the importance of the months during which the pregnant women are in, we treated the months of pregnancy as a confounding factor and adjusted for it in models 3 and 4. Maternal age was also included as a covariate in all models.

Sampling data weighting was applied in all analyses according to the guidelines set by the PMA data analysis manual. Variance inflation factor (VIF) was used to assess multicollinearity among independent variables, but all had a VIF value of less than three, which was below the generally accepted cut-off value of five [25]. All data analyses were conducted using Stata 16.1.

### Ethics and consent

The PMA received ethical approval from Addis Ababa University, College of Health Sciences (Ref: AAUMF 01–008), and the Institutional Review Board (FWA00000287) of Johns Hopkins University Bloomberg School of Public Health. Informed verbal consent was obtained from the study participants before data collection **[**26**]**.

## Results

### Sample characteristics

Table 1 displays the characteristics of the respondents, as well as their preferences for home delivery. The mean age of the participants was 27 years and the median parity was two children. Of 678 participants, approximately two-thirds were between the ages of 20–34 years (70%) and 44% never attended formal school. The majority of the participants lived in rural areas (77%) and developed regions (88%). More than half of the participants (58%) were multipara, 11% had attended ANC by HEWs, and 37% by skilled health professionals. Furthermore, 54% had discussed the place of delivery with their partners for the current pregnancy and 4% participated in ‘1 to 5’ meetings. Concerning community support, 48% of the pregnant women perceived high community support for facility delivery.

**Table 1.** Sample characteristics of the respondent women in Ethiopia (n = 678) and their preferences for home delivery.

Characteristics	Weighted Frequency (%)	Preference for Home Delivery Weighted Frequency (%)
Total	678 (100)	226 (33)
**Individual factors**		
Age group		
>34	126 (19)	53(42)
20–34	476 (70)	142 (30)
15–19	76(11)	31(41)
Education		
Secondary or higher	133 (20)	13 (10)
Primary education	246 (36)	77 (31)
Never attended	299 (44)	136 (45)
Residence		
Urban	155 (23)	15 (9)
Rural	523 (77)	211(40)
Region		
Developed	596 (88)	192 (32)
Emerging	54 (8)	31 (57)
City administrations	28 (4)	3 (11)
Media Exposure		
Yes	250 (37)	62 (24)
No	428 (63)	164 (39)
Wealth quantile		
Highest quantile	127 (19)	9 (7)
Higher quantile	101 (15)	26 (26)
Middle quantile	135 (20)	55 (41)
Lower quantile	152 (22)	66 (44)
Lowest quantile	163 (24)	70 (43)
Parity		
Nullipara	169 (25)	34 (20)
Primipara	113 (17)	28 (25)
Multipara	396 (58)	164 (41)
**Household-supporting factors**		
Partner support for ANC		
Yes	500 (74)	119 (24)
No	178 (26)	107 (61)
Discussed with a partner about the place of delivery		
Yes	366 (54)	71 (19)
No	312 (46)	155 (50)
**Healthcare factors**		
ANC visit in the current pregnancy		
ANC by skilled health professionals	250 (37)	50 (20)
ANC by HEWs	76 (11)	23 (30)
No ANC	352 (52)	153 (43)
**Community-supporting factors**		
Participated in ‘1 to 5’ meeting		
Yes	30 (4)	2 (6)
No	648 (96)	224 (35)
Community support		
High support	324 (48)	48 (14)
Low support	354 (52)	178 (55)

### Preference for home delivery

As shown in Table 1, out of the 678 pregnant women included in the study, 226 (33%) showed a preference for home delivery. Among women >34 and 15–19 years old, 40% and 41% preferred to deliver at home, respectively. Similarly, 45% of women who never attended formal school and 10% of those who attended secondary school or higher showed home delivery preference. Pregnant women living in rural areas (40%) and emerging regions (57%) showed a higher preference for home delivery, compared to those living in urban areas (9%) and city administrations (11%). Moreover, 7% of women from the highest quantile of wealth and 43% from the lowest quantile preferred to deliver at home. Among the nulliparas and primiparas, 20% and 25% preferred to deliver at home, respectively.

Forty-three percent of women who had no ANC visit in the current pregnancy and 30% of those who had ANC visit by HEWs showed home delivery preference. A larger proportion of women who did not have partner support for ANC (61%) and did not discuss the place of delivery with their partners (50%) showed a preference for home delivery, compared to those who had partner support for ANC (24%) and those who discussed the place of delivery with their partners (19%), respectively. Approximately one-third (35%) of women who did not participate in ‘1 to 5’ meetings and 6% of those who participated also showed home delivery preference. Moreover, a larger proportion of women who perceived low community support for facility delivery (55%) preferred home delivery compared to those who perceived high community support (14%).

### Factors associated with preference for home delivery

In model 1 (bivariate analysis), all the independent variables were significantly associated with pregnant women’s preferences for home delivery. When adjusted for individual, household-supporting, and healthcare factors in models 2 and 3, age, residence, region, parity, discussing with a partner about the place of delivery and ANC in the current pregnancy continued to be statistically significant. In the final model, residence and region lost their significance, while participating in the ‘1 to 5’ meetings and community support became statistically significant (Table 2).

**Table 2.** Bivariate and multivariate analysis of factors associated with pregnant women’s preferences for home delivery in Ethiopia.

	Model 1 PR (95% CI)	Model 2 Adj. PR (95% CI)	Model 3 Adj. PR (95% CI)	Model 4	
				Adj. PR (95% CI)	P-value
**Individual factors**					
Age					
>34	1	1	1	1	
20–34	0.7 (0.52–0.96) *	1.0 (0.77–1.33)	1.1 (0.86–1.44)	1.0 (0.83–1.36)	0.59
15–19	0.9 (0.64–1.52)	2.3 (1.33–4.01) *	2.6 (1.51–4.47) *	2.3 (1.43–4.00) *	**<0.01**
Education					
Secondary or higher	1	1	1	1	
Primary education	3.1 (1.64–5.90) *	1.5 (0.78–2.93)	1.2 (0.67–2.45)	1.1 (0.61–2.20)	0.63
Never attended	4.5 (2.45–8.49) *	1.5 (0.82–3.07)	1.3 (0.70–2.57)	1.3 (0.70–2.49)	0.38
Residence					
Urban	1	1	1	1	
Rural	4.3 (2.49–7.70) *	2.1 (1.11–4.00 *	2.0 (1.14–3.82) *	1.7 (0.98–3.10)	0.05
Region					
Developed region	1	1	1	1	
Emerging region	1.7 (1.33–2.38) *	1.6 (1.29–2.04) *	1.3 (1.10–1.72) *	1.1 (0.94–1.43)	0.14
City administrations	0.3 (0.15–0.77) *	1.7 (0.62–5.04)	1.7 (0.61–4.77)	1.6 (0.59–4.53)	0.33
Media exposure					
Yes	1	1	1	1	
No	1.5 (1.15–2.15) *	0.9 (0.73–1.33)	0.9 (0.69–1.20)	0.8 (0.64–1.09)	0.20
Wealth Index					
Highest quantile	1	1	1	1	
Higher quantile	3.8 (1.72–8.56) *	1.4 (0.65–3.32)	1.4 (0.66–3.33)	1.4 (0.63–3.22)	0.38
Middle quantile	6.0 (2.95–12.39) *	2.0 (0.93–4.66)	2.1 (0.94–4.71)	2.1 (0.95–4.68)	0.64
Lower quantile	6.5 (3.24–13.38) *	1.7 (0.79–4.07)	1.7 (0.78–4.03)	1.7 (0.78–4.01)	0.16
Lowest quantile	6.3 (3.14–12.84) *	1.6 (0.72–3.65)	1.7 (0.76–3.82)	1.6 (0.75–3.73)	0.20
Parity					
Nullipara	1	1	1	1	
Primipara	1.2 (0.73–2.11)	1.5 (0.88–2.55)	1.3 (0.77–2.20)	1.4 (0.88–2.32)	0.14
Multipara	2.0 (1.37–3.06) *	2.0 (1.25 3.31) *	1.8 (1.15–3.01) *	1.8 (1.19–2.92) *	**<0.01**
**Household-supporting factors**					
Partner support for ANC					
Yes	1	1	1	1	
No	2.5 (2.01–3.27) *	1.3 (1.06–1.83) *	1.2 (0.94–1.59)	1.0 (0.84–1.38)	0.51
Discussed with a partner about place of delivery					
Yes	1	1	1	1	
No	2.5 (1.91–3.43) *	1.7 (1.28–2.39) *	1.7 (1.29–2.37) *	1.5 (1.18–2.10) *	**<0.01**
**Healthcare factors**					
ANC visit in the current pregnancy					
ANC by skilled health professionals	1	1	1	1	
ANC by HEWs	1.5 (0.88–2.58)		1.1 (0.71–1.88)	1.3 (0.88–2.04)	0.16
No ANC visit	2.1 (1.52–3.05) *		1.7 (1.22–2.39) *	1.5 (1.11–2.11) *	**<0.01**
**Community supporting factors**					
Participated in ‘1 to 5’ meetings					
Yes	1			1	
No	6.1 (1.27–29.79) *			4.5 (1.09–18.95) *	**0.03**
Community support					
High support	1			1	
Low support	4.0 (2.82–5.82) *			2.2 (1.53–3.20) *	**<0.01**

Note: PR, crude prevalence ratio; Adj. PR, adjusted prevalence ratio; CI, confidence interval *P < 0.05

Pregnant women between the ages of 15–19 years (PR = 2.3; 95% CI: 1.43–4.00) and multiparas (PR = 1.8; 95% CI 1.19–2.92) were more likely to prefer home delivery compared to their references. Similarly, pregnant women who did not discuss place of delivery with their partners (PR = 1.5; 95% CI: 1.18–2.10), those who had no ANC visit in the current pregnancy (PR = 1.5: 95% CI: 1.11–2.11), those who did not participate in the ‘1 to 5’ meetings (PR = 4.5; 95% CI: 1.09–18.95), and those who perceived low community support for health facility delivery (PR = 2.2; 95% CI: 1.53–3.20) were more likely to prefer home delivery, compared to their reference group.

## Discussion

This study described pregnant women’s preferences for home delivery and examined individual, household-supporting, healthcare, and community-supporting factors associated with this preference. Thirty-three percent of pregnant women showed home delivery preference. This study also identified that younger age, multiparity, not attending ANC in the current pregnancy, not discussing the place of delivery with a partner, not participating in ‘1 to 5’ meetings, and perceived low community support appear to be associated with home delivery preference among pregnant women in Ethiopia.

The prevalence of home delivery preference is consistent with a study conducted in Nigeria [27] but higher than the results of a study conducted in the town of Debre Markos in Ethiopia in which 19.6% of pregnant women showed a preference for home delivery [16]. A possible explanation lies in the national versus local study populations captured in these respective studies. Moreover, the finding of this study showed a lower preference for home delivery, compared to the 2019 EDHS report in which 51% of women delivered at home [3]. This difference may be due to a potential response bias, as well as the reason that even if many women perceive the need to deliver at a health facility during their pregnancy periods, they may end up delivering at home, due to reasons such as the sudden onset of labour and lack of transportation to the health facility [16].

In contrast to other studies [28], pregnant women between the age of 15–19 years showed a higher preference for home delivery. In our perspective, this may be due to the reason that since most women in these age groups may not have experiences with childbirth, they may prefer to deliver at home thinking that they will receive more support from their family. In addition, women of these age groups may not be in the position to decide for themselves because they are often financially dependent on their parents or partners, which may also influence their delivery place preferences [29]. In order to increase understanding of these issues, further research is recommended.

This study also showed that being multipara is associated with home delivery preference in Ethiopia. This finding is consistent with other studies conducted among postnatal women in Ghana, and Ethiopia [11,13]. A possible explanation may be that, compared to the nulliparas, multiparas are usually confident regarding delivery because of their previous childbirth experiences [30].

Not attending ANC visits in the current pregnancy is also associated with a preference for home delivery in Ethiopia. This finding is consistent with studies conducted in Nigeria and Ethiopia [27,31,32]. It could be that women who had ANC visits by health professionals had better opportunities to discuss and be counseled regarding birth preparedness, complication readiness, and place of delivery [31].

According to this study, pregnant women who did not discuss place of delivery with their partners had a higher risk of home delivery preference, compared to those who did discuss it. Prior studies conducted in Tanzania and Mozambique have shown similar results [33]. There are several possible explanations for this finding. For example, if the pregnant woman is financially dependent on her partner, involving and discussing the place of delivery may improve her confidence in choosing the preferred place of delivery [34]. Furthermore, husbands who have positive attitudes towards facility deliveries may encourage their wives to deliver at facilities during their discussions [35]. However, approximately half the participants of our study did not discuss delivery alternatives with their partners, possibly because they were not sufficiently empowered to initiate such discussions. This may be due to the cultural influences present in most rural as well as the emerging regions of Ethiopia where husbands, clan members, mothers/mothers-in-law, and traditional birth attendants are the main decision-makers in the women’s place of delivery [36].

Not participating in the ‘1 to 5’ meetings had the highest effect size for home delivery preference of all the determinants. This finding has also been observed in a study conducted among postnatal women in Hamar, Ethiopia, which showed that women who did not participate in the WDA had a higher risk for home delivery compared to those who participated [32]. Women who participated in the ‘1 to 5’ meetings of the WDA may share their knowledge and experience on previous health facility deliveries, with women of other households on their team as well as help to strengthen their decision-making authority within their households [21], influencing pregnant women decision on facility delivery.

This study also revealed that perceived low community support to be associated with home delivery preference in Ethiopia. A prior study conducted in the Tigray region of Ethiopia identified that traditional factors, such as community norms, values, and religious beliefs, impact women’s decisions regarding their chosen places of delivery [35]. According to the same study, elderly women and traditional birth attendants in the Tigray region, who are highly respected in the community, actively encouraged mothers towards home delivery [35].

Prior studies have identified factors such as perceived poor quality of healthcare services, unwelcoming attitudes of health professionals, home deliveries as customary practice in society as well as distance and poor access to transportation as reasons for home delivery in Ethiopia [28]. These factors may also adversely influence pregnant women’s attitudes towards health facility delivery encouraging them to prefer home delivery.

### Strengths and limitations of the study

The analyses were performed on data from a community-based national survey of pregnant women living in both urban and rural settings in Ethiopia, including a high response rate decreasing the selection bias. This is the first study assessing preference for home delivery with national coverage in the country.

However, some limitations should be considered. First, the PMA cross-sectional survey data used a sample size estimation based on the prevalence of modern contraceptive use from previous studies rather than on the prevalence of home delivery preference, which led to a smaller study sample. Second, the questionnaire used by the PMA assessed pregnancy status through the women’s self-report of their pregnancies and this may have resulted in missing early pregnancies. Third, some relevant factors such as the place of antecedent pregnancy and the outcomes could not be explored in this study due to availability of the data. Since these factors might be related to women’s preference for home delivery, their inclusion could have affected our findings. Finally, because of the self-reported questionnaire, response bias may be operating. The role of these two biases could not, however, be determined.

## Conclusions

In conclusion, a large number of pregnant women still preferred home for deliveries in Ethiopia. Household factors, such as not discussing place of delivery with a partner, and a lack of community supporting factors, had the most influence on this preference.

In order to reduce these preferences for home delivery, efforts should be made particularly to strengthen the ‘1 to 5’ meetings as part of the women’s developmental army activities at the community level. Awareness regarding the risks associated with home deliveries should be reinforced in the messaging to the community using different media sources and community-based programmes. Strategies to educate young women’s parents and partners regarding the importance of discussing the preferred place of delivery should also be implemented in the country.

## Paper context

Although prior studies retrospectively assessed factors associated with home deliveries in Ethiopia, no study explored risk factors associated with pregnant women’s preference for home delivery. This study investigates the individual, household, healthcare, and community-supporting factors associated with home delivery preferences in Ethiopia. Efforts should be taken to strengthen community interventions such as the ‘1 to 5’ meetings and develop strategies to educate young women’s parents and partners regarding the importance of discussion about the place of delivery.
